# Supplementary material for: Lysophosphatidic Acid Receptor 3 Activation Is Involved in the Regulation of Ferroptosis
Source: Int J Mol Sci. 2024 Feb 15;25(4):2315. doi: 10.3390/ijms25042315 (PMC10889550; doi:10.3390/ijms25042315)
Supplement: Supplementary file 1 [file ijms-25-02315-s001.zip › ijms-2837242-supplementary.pdf]

Figure S1. Western blot images developed by UVP illuminator

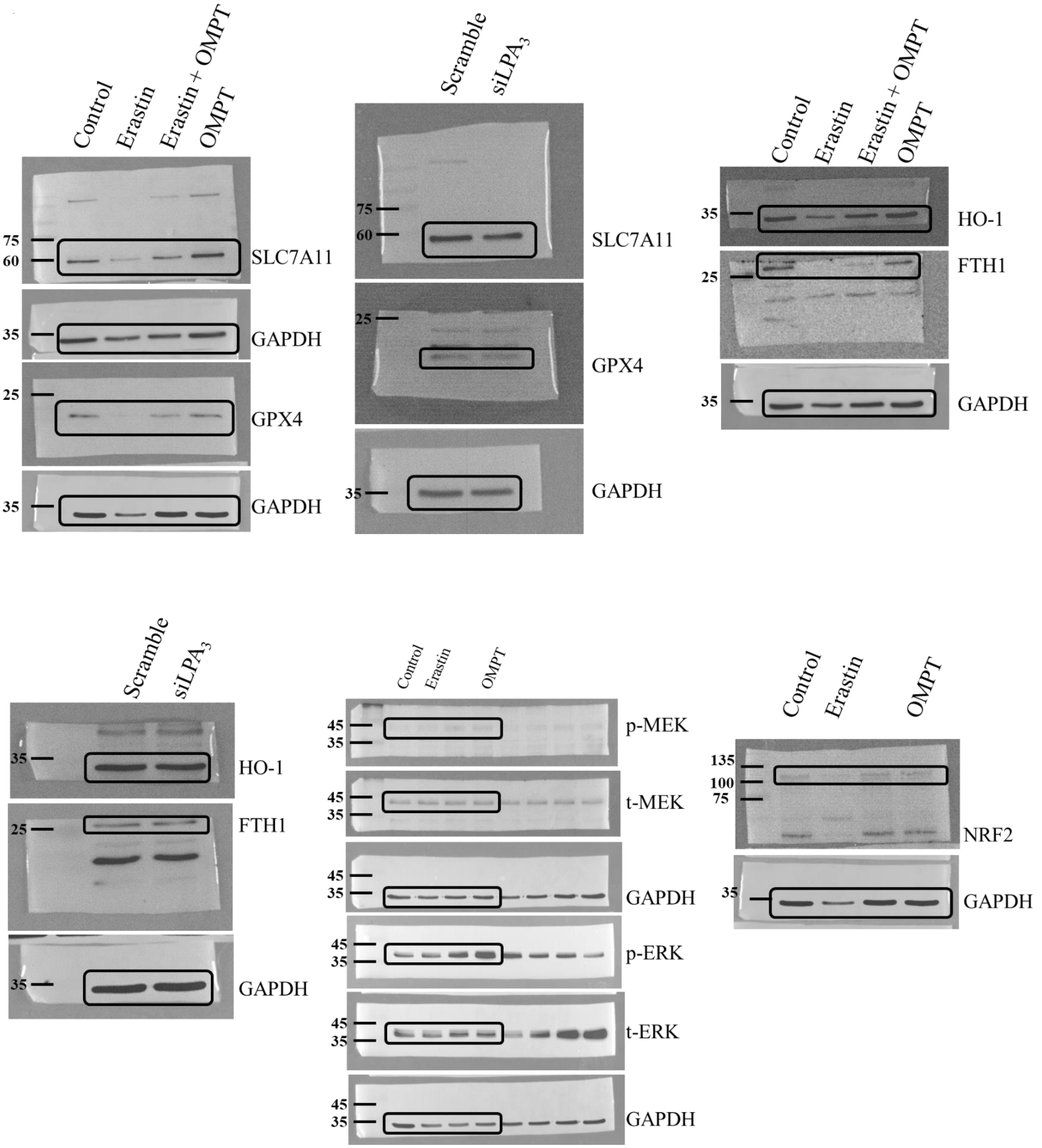

Table S1. Western blot analyses of LPAR3 knockdown

SLC7A11

Table Analyzed	SLC7A11	Scramble	siLPA$_3$
		1	0.780741
Column B	siLPA$_3$	1	0.89553
vs.	vs.	1	0.700098
Column A	Scramble	1	0.888689
Unpaired t test			
**P value**	**0.0077**		
**P value summary**	**		
**Significantly different (P < 0.05)?**	**Yes**		
One- or two-tailed P value?	Two-tailed		
t, df	t=3.926, df=6		
How big is the difference?			
Mean of column A	1		
Mean of column B	0.8163		
Difference between means (B - A) ± SEM	-0.1837 ± 0.04680		
95% confidence interval	-0.2983 to -0.06922		
R squared (eta squared)	0.7198		
F test to compare variances			
F, DFn, Dfd	Infinity, 3, 3		
P value	<0.0001		
P value summary	****		
Significantly different (P < 0.05)?	Yes		
Data analyzed			
Sample size, column A	4		
Sample size, column B	4		

Table S2. Western blot analyses of LPAR3 knockdown

GPX4

Table Analyzed	GPX4	Scramble	siLPA$_3$
		1	0.90336
Column B	siLPA$_3$	1	0.914376
vs.	vs.	1	0.852389
Column A	Scramble		
Unpaired t test			
**P value**	**0.0045**		
**P value summary**	**		
**Significantly different (P < 0.05)?**	**Yes**		
One- or two-tailed P value?	Two-tailed		
t, df	t=5.759, df=4		
How big is the difference?			
Mean of column A	1		
Mean of column B	0.89		
Difference between means (B - A) ± SEM	-0.1100 ± 0.01909		
95% confidence interval	-0.1630 to -0.05695		
R squared (eta squared)	0.8924		
F test to compare variances			
F, DFn, Dfd	Infinity, 2, 2		
P value	<0.0001		
P value summary	****		
Significantly different (P < 0.05)?	Yes		
Data analyzed			
Sample size, column A	3		
Sample size, column B	3		

Table S3. Western blot analyses of LPAR3 knockdown

FTH1

Table Analyzed	FTH1	Scramble	siLPA$_3$
		1	0.803041
Column B	siLPA$_3$	1	0.702253
vs.	vs.	1	0.808402
Column A	Scramble	1	0.639329
		1	0.836733
Unpaired t test			
**P value**	**0.0002**		
**P value summary**	*******		
**Significantly different (P < 0.05)?**	**Yes**		
One- or two-tailed P value?	Two-tailed		
t, df	t=6.474, df=8		
How big is the difference?			
Mean of column A	1		
Mean of column B	0.758		
Difference between means (B - A) ± SEM	-0.2420 ± 0.03739		
95% confidence interval	-0.3283 to -0.1558		
R squared (eta squared)	0.8397		
F test to compare variances			
F, DFn, Dfd	Infinity, 4, 4		
P value	<0.0001		
P value summary	****		
Significantly different (P < 0.05)?	Yes		
Data analyzed			
Sample size, column A	5		
Sample size, column B	5		

Table S4. Western blot analyses of LPAR3 knockdown

HO-1

Table Analyzed	HO-1	Scramble	$siLPA_3$
		1	0.842527
Column B	$siLPA_3$	1	0.915276
vs.	vs.	1	0.912908
Column A	Scramble	1	0.887303
		1	0.843381
Unpaired t test			
**P value**	**<0.0001**		
**P value summary**	********		
**Significantly different (P < 0.05)?**	**Yes**		
One- or two-tailed P value?	Two-tailed		
t, df	t=7.479, df=8		
How big is the difference?			
Mean of column A	1		
Mean of column B	0.8803		
Difference between means (B - A) ± SEM	-0.1197 ± 0.01601		
95% confidence interval	-0.1566 to -0.08281		
R squared (eta squared)	0.8749		
F test to compare variances			
F, DFn, Dfd	Infinity, 4, 4		
P value	<0.0001		
P value summary	****		
Significantly different (P < 0.05)?	Yes		
Data analyzed			
Sample size, column A	5		
Sample size, column B	5		
